# Perceptions and Experiences of Human Papillomavirus (HPV) Infection and Testing among Low-Income Mexican Women

**DOI:** 10.1371/journal.pone.0153367

**Published:** 2016-05-05

**Authors:** Leith León-Maldonado, Emily Wentzell, Brandon Brown, Betania Allen-Leigh, Leticia Torres-Ibarra, Jorge Salmerón, Deborah L. Billings, James F. Thrasher, Eduardo Lazcano-Ponce

**Affiliations:** 1 CONACYT, Instituto Nacional de Cancerología, Ciudad de México, México; 2 Centro de Investigación en Salud Poblacional, Instituto Nacional de Salud Pública, Cuernavaca, Morelos, México; 3 Department of Anthropology, The University of Iowa, Iowa City, IA, United States of America; 4 Department of Social Medicine and Population Health, School of Medicine, University of California, Riverside, CA, United States of America; 5 Unidad de Investigación Epidemiológica y en Servicios de Salud, Instituto Mexicano del Seguro Social, Cuernavaca, Morelos, México; 6 Department of Health, Promotion, Education and Behavior, Arnold School of Public Health, University of South Carolina, Columbia, SC, United States of America; Rudjer Boskovic Institute, CROATIA

## Abstract

**Background:**

HPV infection causes cervical cancer, a major contributor to morbidity and mortality among low-income Mexican women. Human papillomavirus (HPV) DNA testing is now a primary screening strategy in Mexico’s early cervical cancer detection program (ECDP). Research on Mexican women’s perceptions of HPV and testing is necessary for establishing culturally appropriate protocols and educational materials. Here, we explore perceptions about HPV and HPV-related risk factors among low-income Mexican ECDP participants.

**Methods:**

We conducted semi-structured interviews with 24 ECDP participants from two primary care health clinics in Michoacán state, Mexico. Interviews addressed women’s understandings of and experiences with HPV and HPV testing. Analysis was inductive and guided by the Health Belief Model with a focus on gender.

**Results:**

Women’s confusion about HPV and HPV screening caused emotional distress. They understood HPV to be a serious disease that would always cause severe symptoms, often characterizing it as analogous to HIV or inevitably carcinogenic. Women also attributed it to men’s sexual behaviors, specifically infidelity and poor hygiene. Women described both sexes’ desire for sex as natural but understood men’s negative practices of masculinity, like infidelity, as the causes of women’s HPV infection. Some women believed dirty public bathrooms or heredity could also cause HPV transmission.

**Conclusions:**

These results are consistent with prior findings that geographically and economically diverse populations lack clear understandings of the nature, causes, or symptoms of HPV, even among those receiving HPV testing. Our findings also reveal that local cultural discourse relating to masculinity, along with failure to provide sufficient education to low-income and indigenous-language speaking patients, exacerbate women’s negative emotions surrounding HPV testing. While negative emotions did not deter women from seeking testing, they could be ameliorated with better health education and communication.

## Introduction

Cervical cancer is one of the main causes of morbidity and mortality among Mexican women, especially low-income women, despite the presence of a national early cervical cancer detection program (ECDP) since the 1970s. [1–4] In 2008, this program switched from cytology to HPV testing as the primary cervical cancer screening tool.[5,6] This screening strategy is promising since HPV testing is more sensitive than cervical cytology for detecting high-degree lesions, is more cost-effective than cytology, and is more feasible than cytology in developing countries that face economic and organizational barriers to comprehensive cytology-based screening programs.[1,7–14]

Despite the benefits that HPV testing offers as a primary ECDP screening tool, the concept of cervical cancer and screening as virus-related have been found to cause some negative psychosocial effects. Women being tested for or diagnosed with HPV may experience anguish, anxiety, confusion, fear or stigma.[15–19] These experiences might deter women from undergoing HPV testing and cervical cancer screening.[20,21]

Such perceptions and experiences regarding HPV and other sexually-transmitted infections (STIs) are profoundly shaped by local culture, especially norms and beliefs regarding sexuality and gender.[17,22] In Mexico, STIs are often stigmatized, posing a risk of emotional suffering that might outweigh their biological risk.[23] Popular cultural understandings of STIs are also powerfully linked to gender norms, including nationwide critique of “macho” masculinities characterized by infidelity,[24,25] and local patterns of male work migration such as the large-scale male migration that occurs in Michoacán.[26]

For these reasons, it is important to assess women’s beliefs about and experiences of HPV in diverse local contexts, in order to understand how they might influence women’s screening experiences. This information is also important for developing educational materials and culturally-sensitive screening protocols, which would increase ECDP use and mitigate any psychosocial harm that screening might pose. Here, we focus on the ECDP experiences of low-income Mexican women in marginalized areas of Michoacán state.

## Methods

### Ethics statement and informed consent procedure

All participants provided verbal informed consent, which we employed since low levels of education and literacy in the study population made written consent inappropriate. Consent was obtained through the following procedure. The principal investigator read the letter of informed consent to each potential participant, which included a description of the study including its objectives, potential risks and benefits, the voluntary nature of participation and participants’ ability to withdraw at any time. Potential participants were also given an information card that listed the study title, names, titles and contact information for the ethics board members and study principal investigator, and a statement that the participant could call and ask for information or make comments or complaints. The principal investigator also read this card aloud to potential participants. Women who wished to participate then gave verbal consent, which was audio recorded. As mandated by the local ethics board, this procedure occurred in the presence of at least one witness. This study, including the verbal consent procedure and script followed, was approved by the Mexican National Institute of Public Health Ethics Board (IRB).

### Study Setting and Participants

We conducted semi-structured interviews with women who underwent HPV testing in the ECDP in order to explore their beliefs and perceptions regarding HPV. Participants were ECDP service users drawn from the patient pools of two primary health care centers in the Michoacán State Health Service. These two health centers were selected because they were located in low-income municipalities but served populations that varied ethnically. One center was in the state capital of Morelia, and had a Spanish-speaking, mestizo population. The other center was in the town of Chilchota in Northern Michoacán, which had a large indigenous population composed of Purepecha and Tarasco speakers who had experienced a long history of marginalization from state and economic offerings.[27] We drew participants from both locations in order to assess whether marginalization based on indigenous status influenced women’s perceptions of HPV in Michoacán state. [28]

### Procedures

From each municipal health care center, we received a list of women who had undergone HPV testing and were given results within the past month. We then verbally invited those women to participate, stopping after 12 women from each site had agreed to participate. Invitations were given in one of three ways: in person at a woman’s next clinical appointment if she had one scheduled during the recruitment period, over the telephone, or at her home if she could not be reached by phone and did not have an appointment scheduled. We explained the purpose of the study, the fact that her participation was voluntary, and that her information would be confidential. We also described the study aims and methods. We then explained the study and collected verbal informed consent from women who wished to participate, as discussed above. Interviews with consenting participants subsequently took place in private rooms at the clinical sites, with the exception of three which were held in private spaces in the homes of Morelia participants for their convenience.

The design of the broader study of women’s and healthcare providers’ HPV-related experiences, of which this research forms a part, is described in detail elsewhere.[29] Participants in the present analysis responded to an interview guide administered in Spanish by a Mexican female investigator trained in qualitative research methods (Table 1). In the semi-structured interview format, open-ended questions served as starting points from which participants could add additional information and introduce new themes.

**Table 1:** Semi-structured interview questions.

***HPV testing motivations***
Why did you decide to get the HPV test?
Why did you think it was necessary to do it?
Would you recommend the HPV test to a friend, relative or neighbor? Why?
In your view, why do women need to get the HPV test?
How would you convince someone to get the HPV test? What would you say to motivate her?
Does a woman’s partner influence her decision to get these tests? How and why?
Why do you think that women don’t get the HPV test?
***Experience of receiving HPV test results***
How was getting the results? What did they (health professionals) tell you?
Did they explain the results in a way that was easy to understand?
What else would you have liked them to explain?
Could you ask questions? Did you ask any questions?
Do you still have questions?
Can you think of anything you would have liked to ask?
How did you feel while getting the results?
Was there anything you didn’t like when they gave you your results? Anything you did like?
What does the HPV result you got mean to you? What does positive or negative mean?
Would you like to receive information on that topic? How would you like to receive that information?
***Comparing HPV and Pap testing experiences***
Is it worth it to have a cervical cancer screening test? Why?
What would you have liked to be different?
Did they give you the Pap (cervical cytology) and the HPV test?
How often do you get a Pap test? Where do you get it?
Which did you like better, the HPV test or the Pap?
If you could choose between the HPV and Pap test, which would you get? Why?
What didn’t you like about that test?
Do you think there are differences between the tests? What are the differences?
When they did the Pap test, did anything worry you? How did you feel, during the test? And when you received the results?
And now that they did the HPV test, has anything worried you?
***Perceived need for testing***
Which women do you think should worry about the Pap test? Which women should have it done?
Which women do you think should worry about the HPV test? Which women should have it done?
Do you think that women’s sex live have anything to do with whether they need cervical cancer detection tests? With needing the Pap? With needing the HPV test?
***Improving the testing experience***
What should they do to encourage women to come get the HPV test?
What could they do here in the unit to make it easier for women to get the HPV test?
What could they do here in the unit to make your next HPV test experience better? What could they do to make it easier for you next time?
Is there anything that I haven’t asked that you would like to share?

Questions collected sociodemographic data and assessed participants’ perceptions and phenomenological experiences of HPV and HPV testing. Question design was guided by the Health Beliefs Model (HBM), which indicates that experiences of vulnerability (in this case, perceptions of the physical and social risks of HPV) and perceptions of severity or seriousness of an event (here, the perceived implications of HPV infection) together influence adoption of preventative behavior (in this case, undergoing screening).[30] Interviews lasted half an hour on average, and were audio recorded with last names excluded. Recordings were subsequently transcribed by trained transcriptionists, and the authors translated the quotations presented here into English.

### Data analysis

Our analytic aim was to assess participants’ perceptions and experiences of HPV and HPV testing in the context of local culture, in relationship to the components of the HBM model described above. Data analysis was inductive, meaning that it was guided by the specifics of the data collected rather than a hypothesis based on our theoretical model. The sample size of 24 participants, with 12 from each site, was sufficient for achieving data saturation in a thematic analysis framework, specifically for identifying themes common to multiple participants and discerning whether thematic differences existed between the two sites.[31] We identified emerging themes in interview transcripts related to HPV vulnerability, perceptions of HPV severity/seriousness, and gender since the latter, while not a component of the HBM model, was a major theme in participants’ responses. We then developed a code list that reflected those themes and coded the transcripts using the ATLAS.ti program, collaboratively assessing the codes and coding for validity and reliability throughout the process. We then organized data related to the themes we had identified into tables, and identified patterns that emerged between participants’ experiences (such as spousal infidelity or receipt of written test results) and their stated understandings of HPV and HPV testing (such as beliefs about HPV symptoms and transmission).[32]

## Results

**Fig 1:**
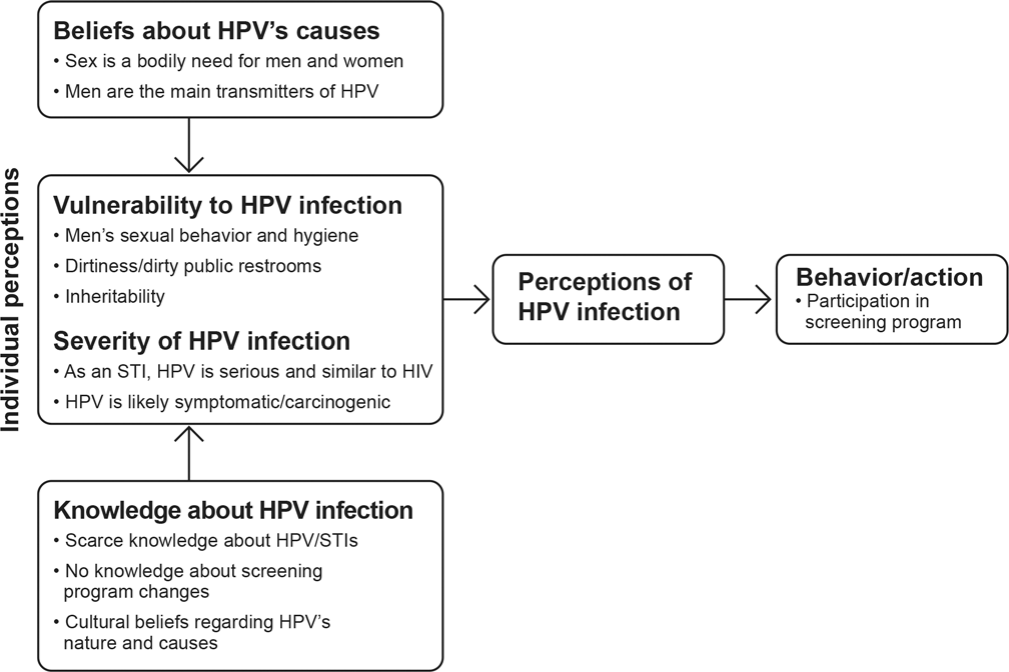
Beliefs, perceptions and behavior regarding HPV infection among low-income Mexican women. HPV = human papillomavirus, STI = sexually transmitted infection, HIV = human immunodeficiency virus.

Here, we provide an overview of participant demographics, then present the key themes that arose in women’s interviews, exemplified with representative quotations. Two main kinds of results emerged from the data, regarding women’s beliefs about the nature and severity of HPV, and their perceptions of HPV’s causes and risks (Fig 1. Beliefs, perceptions and behavior regarding HPV infection among low-income Mexican women. HPV = human papillomavirus, STI = sexually transmitted infection, HIV = human immunodeficiency virus.).

### Participant demographics

A total of 24 ECDP users, 12 attending each health center, participated in the study. Inclusion criteria were: being a woman who received HPV testing as primary cervical cancer screening in the ECDP, receiving HPV test results in the month prior to study recruitment, and having enough Spanish language fluency to participate in the consent process and interview. All participants invited agreed to participate, except for two who cited lack of time. Participant demographics are described in Table 2. Briefly, they had an average age of 45 years. Most were married, had children, and had attended 6 or fewer years of school. Almost half identified as homemakers. Most were beneficiaries of the Oportunidades Program (modified into the Prospera Program after our data collection), a federal cash-transfer program intended to raise access to social and health services and increase economic security. [33] Almost half also subscribed to Seguro Popular, a low-cost public health insurance system for Mexican citizens who lack access to employment-based national health care systems.[34] Of the 24 participants, six women received a positive HPV test result. Participants had not received health education or counseling regarding HPV, aside from the clinical explanation that positive HPV test results would require further follow-up.

**Table 2:** Socio-demographic and clinical characteristics of participants.

Characteristics	Participants	
	Chilchota (n = 12)	Morelia (n = 12)
**Age groups (years)**		
30–39	1	6
40–49	9	2
50–59	0	4
60–64	1	0
≥65	1	0
**School (years)**		
None	1	0
<6	6	3
6	4	5
≥7	1	4
**Marital status**		
Single	0	1
Married/Cohabitating	10	9
Divorced/separated/widowed	2	2
**Occupation type**		
Housewife	4	8
Business employee	5	5
Housekeeper	0	1
Independent vendor	3	0
**Indigenous language speaker**		
Yes	11	0
No	1	12
**Oportunidades program recipient**		
Yes	12	7
No	0	5
**Births**		
0	1	0
1–3	3	6
4–6	6	5
≥7	2	1
**HPV testing result**		
Positive	6	0
Negative	6	12

Almost half of the participants, all at the Chilchota site, reported that they spoke an indigenous language. The only women who received positive HPV diagnoses were indigenous women at the Chilchota site. There were no other major differences in the data from the two sites or from the clinic- versus home-based interviews.

### Key themes present in the interviews

In this section we present the key themes that emerged from the interviews, together with representative quotations (Table 3).

**Table 3:** Key themes.

Theme	Subtheme	Example
**Confusion about HPV leads to fear**	***Lacking HPV knowledge***	“I don’t know what this disease is, I’ve just seen on TV that it affects many women, but I don’t understand how or what it does” *(Chilchota participant)*.
		Wanting doctors “to explain clearly what exactly the human papillomavirus is, because people don’t really know what the human papillomavirus is, including me” *(Morelia participant)*.
	***Assuming HPV is serious and/or is the same as cervical cancer or like HIV/AIDS***	“What do I have? Is it serious? I was scared and I thought it will probably be serious, who knows what will happen…They gave me the positive result…I think it’s probably serious” *(Chilchota participant)*.
		People with HPV “feel really sick, sometimes they bleed, sometimes they itch or they feel bad” *(Morelia participant)*.
		“I’ve only heard on TV that that people who have [HPV]…they get cancer in their uterus and they die” *(Morelia participant)*.
		HPV is “like AIDS or something…“causes warts and infections and all that, it worried me and I said to myself that I would get [screened] when I saw [the sign publicizing HPV testing]” *(Morelia participant)*.
	***Assumption that HPV testing can reveal more serious consequences than Pap testing***	The participant usually did not “relate the Pap test with death,” but said HPV screening made her think “that it might be AIDS or, I don’t know, I might be really sick” *(Morelia participant)*.
**Fear and confusion regarding HPV testing and results**	***Feeling fear and shame***	“I felt bad…I felt fear that they’d tell me I had cancer or something in my uterus…they say that’s really dangerous and that’s why I was scared” *(Morelia participant)*.
		“I’m just a little scared…because we don’t know what to do with this [positive test result] or where they’ll send me…I want to know, what is this and where did it come from?” *(Chilchota participant)*.
		“No, I didn’t like it [HPV testing]… It made me feel embarrassed” *(Chilchota participant)*.
		“There are people who don’t like to go [to the clinic] to get the tests…it isn’t pleasant but it’s necessary… [Women don’t come] because they’re embarrassed, more than anything because in those [rural] places there’s lots of people who it really embarrasses…I’m not sure if it’s because of fear or shame, they don’t want to go … it could be because of ignorance, lots of ignorance.” *(Chilchota participant)*.
	***Language barriers increase confusion***	Wanting doctor to explain, “Am I ok? Or, what’s going on, because I don’t know how to read…or write…and I can’t speak well in Spanish. I want to know what’s going on…Am I well or sick?” *(Chilchota participant)*.
	***Believing HPV testing will hurt***	“I would rather that they hurt me, to take care of my health.” *(Morelia participant)*.
	***Confusion about the notification system generates fear***	Woman was told simply “…that the result isn’t good, that we have to do it again…I don’t understand it” *(Chilchota participant)*.
		Woman said she felt fear about the HPV test but not the Pap test, because “In that one [the Pap] no paperwork came but with this one [the HPV test] a paper arrived.” *(Chilchota participant)*.
	***Clearer information given at colposcopy***	Told at colposcopy “Not to be scared…that it wasn’t cancer or anything serious, but we would have to see how another test came out that we would do another time…Every three years we do the Pap test” *(Chilchota participant)*.
**Belief that sex a bodily need, but problematic sexual behavior and masculinity cause HPV risk**		“There are women who don’t have husbands…Sometimes you go with men because, well, the body needs these things, to have sex with a man…[but this creates risk because] You don’t know what kind of person they are” *(Chilchota participant)*.
		“I’ve had various partners because I’m separated” but heard that HPV risk was “related to the partners that one has had” *(Morelia participant)*.
		“I was expecting something bad to come of [my husband’s infidelity]… because I don’t have confidence in my partner… that he’s going to give me an infection or something like that” *(Morelia participant)*.
**Belief that poor hygiene causes HPV risk**	***Including men’s poor sexual hygiene***	“…before there weren’t so many diseases and women didn’t do these tests, but now the situation has changed. I think there’s more dirtiness [suciedad] in men, so there are more sicknesses in women and we have to get treated early” *(Morelia participant)*.
	***Including dirty bathrooms***	You can get HPV “from public bathrooms, or from a husband who goes around with other women” *(Morelia participant)*.
**Belief that HPV is inheritable**		“I have a sister who came down with human papillomavirus and it got me thinking, and that’s why I decided to get tested. Having a family history. Now I know it’s transmitted by your husband, but you can also inherit it” *(Morelia participant)*.
		“I’ve only had one partner, my husband, but he’s been gone a long time. It’s been almost six and a half years since I’ve had sex.” So, she attributed her HPV positivity to “family history.” *(Chilchota participant)*.

## Discussion

### Confusion about HPV leads to fear

All participants were unsure about the exact nature of HPV. Many hoped that receiving HPV testing would clarify this issue. Women often assumed that since they were receiving testing for HPV, it must be a serious disease. All women interviewed associated HPV infection with physical symptoms such as burning, pain, itching or vaginal discharge. Many women believed that since they were attending a cervical cancer screening program, HPV itself must either be a cancer, be like one, or necessarily cause cancer. Some believed it to be or be similar to HIV/AIDS (an association likely strengthened by the similar Spanish-language acronyms for HPV (VPH) and HIV (VIH)). These ideas led women who had not seen the Pap test as especially daunting to be more frightened by HPV testing, which they associated with potentially severe outcomes. Women’s perceptions that HPV was very severe related to these beliefs about symptoms, and to the similarity that women perceived between HPV and HIV.

### Fear and confusion regarding HPV testing results

Such misunderstandings of HPV led to great stress for many women undergoing screening. Women’s knowledge that the disease was sexually transmitted and their fears that it would inevitably cause distressing symptoms also led participants to believe that many women would not get tested due to embarrassment. For women who were primary speakers of indigenous languages, language barriers added to this confusion and augmented their distress. They especially expressed confusion regarding the basic information they were given, for instance understanding “negative” results to mean harmful results, or not understanding the words “positive” or “negative.” However, as with the woman quoted above who would rather be “hurt” by testing to maintain her long-term health, study participants often said that the emotional discomfort of testing was worth the possibility of arresting terminal illness.

In spite of the perceived benefits of testing, confusing messages from health professionals regarding notification procedures and test results made the process emotionally painful for many women. In the ECDP before the introduction of HPV testing, women were notified only if they required follow-up care for abnormal cytology. With HPV testing, women are notified of positive HPV test results which might or might not indicate the presence of pre-cancer. Despite the fact that positive test results are now less closely linked to the presence of pre-cancer, this difference was not communicated and simply receiving results was thus frightening for participants. This led to experiences like that of a woman in Chilchota, who said that she never used to worry about cytology screening but became concerned about her HPV test because of the method of notification of test results. Previously with cytology testing, not receiving paperwork meant a negative result, but when she underwent HPV testing, “the paper arrived.” Since under the prior system receiving “paperwork” meant that one was positive for cervical cancer, participants took positive HPV results to indicate cervical cancer positivity. Women who received positive HPV test results thus experienced distressing confusion regarding the meanings of those test results and the relationship of HPV to cervical cancer. However, the two participants who had recently undergone colposcopy felt that they had received clear explanations about the relationship of HPV positivity to cancer risk. It appears that the explanations provided during colposcopy were more clear and detailed.

### Participants’ beliefs about HPV transmission

Women most frequently understood HPV to be transmitted by problematic sexual behavior. They described desire for sex, for both men and women, as a natural need. However, they feared their own needs would put them at risk. They understood this risk to be rooted in the bodies of untrustworthy male partners, viewing men’s negative practices of masculinity, like promiscuity and infidelity, as actions through which men acquired HPV and would pass it on to female partners. Participants also understood poor hygiene to be a cause of HPV risk, in ways that were linked to their understandings of dangerous male sexual behavior. Some believed that there were more sexually transmitted infections around than in previous eras, and attributed this development to men’s general dirtiness, which encompassed both lack of sexual scruples and poor hygiene. Several participants also identified dirty public restrooms as an additional possible HPV vector. They noted this in concert with the risk posed by men’s poor sexual hygiene and infidelity; this implied that rumors that HPV can be transmitted by dirty bathrooms are voiced but seen more as a cover for transmission through male infidelity than a serious, independent risk factor.

A few women believed that HPV could be hereditary in addition to sexually transmitted. They often felt concern and sought screening when female family members received positive test results. Since women were not informed that HPV can have a very long incubation period, heritability also seemed like a logical explanation for women who had not had sex for several years, for example while their husbands were working abroad.

### Findings in global and local context

The overall finding that participants understood that HPV was sexually transmitted but were confused about its symptoms and its relationship to cervical cancer and other STIs mirrors results from diverse world regions and social classes, indicating that low knowledge regarding HPV is a global problem.[20,35] Prior studies from similarly diverse populations match the present finding that women often report low receipt and comprehension of information about HPV and HPV testing from health personnel.[36,37] Consistent with studies in diverse populations worldwide, [35,38] study participants assumed that HPV was an inevitably serious disease that might be analogous to HIV or necessarily cause cancer. In the present case, both these misunderstandings about HPV and confusion regarding the testing process itself exacerbated the fears and negative emotions that women worldwide often experience when they learn about the sexually transmitted nature of HPV and cervical cancer etiology.[39–42]

We found that study participants shared very similar beliefs about HPV and perceptions of HPV risk regardless of identifying as mestiza or indigenous, other demographic differences such as marital status, educational level and number of children, or whether or not they had received a positive HPV diagnosis. The majority of women participating in the Oportunidades Program and especially the subset of those women participating in Seguro Popular had more contact with health professionals, since they had access to more extensive health care services than others.[33] However, their experiences regarding and ideas about HPV and HPV testing did not differ from other women’s. Women’s statements that they did not receive clear explanations, and were not offered explanations in the Indigenous languages which some of them spoke, accord with prior findings that class and ethnic differences between doctors and Oportunidades patients foster subtle discrimination or hamper health communication.[43,44] The fact that women who had received colposcopy felt that they had received clearer explanations of HPV reflects inconsistency in the education provided by health workers performing different aspects of the screening program. Overall, our findings suggest that in this context, the quality of health education about HPV and communication between health workers and screening program participants must be improved to enhance low-income Mexican women’s understandings of HPV and improve their psychosocial experiences of HPV testing. For communication to improve, health professionals must also have sufficient knowledge about HPV and comfort discussing it to communicate effectively, and lacks in these areas have been documented worldwide.[45–49]

While testing for and diagnosis of sexually transmitted infections can cause emotional pain and stigma worldwide,[19,41,50,51] local cultural ideas about gender and sexuality shape that experience. While studies from many regions have found that women often view infidelity as a key cause of HPV transmission[52–54], participants in the present study incorporated locally-specific ideas about infidelity as innate to untrustworthy men into their understandings of HPV risk. In Mexico, popular cultural discussions of masculinity involve heated debate; understandings of male infidelity as natural or inevitable conflict with widespread critiques of machismo.[24,25] Some women also incorporated locally circulating ideas about unhygienic bathrooms and hereditary risk into their broader understandings of male “dirtiness” as the primary transmission vector, in ways that helped them understand their own HPV positivity in the context of sex lives interrupted by men’s work migration.

The women interviewed here underwent HPV testing despite their confusion and negative emotions regarding HPV and the testing experience, and their beliefs that others might be too embarrassed to undergo testing. While this study only included women who had undergone testing and thus did not capture the experiences of women who might have refused it, study participants’ fears about HPV severity facilitated rather than hindered their participation. However, their testing and diagnosis experiences were characterized by negative emotions related both to the gendered ideas discussed above, and poor communication by health professionals. This finding suggests that promotion of HPV testing and cervical cancer screening is not enough; culturally- and linguistically-appropriate education about those issues, as well as clearer communication between health workers and screening participants about these topics, is needed to increase screening among low-income Mexican women by making it a more positive experience.

### Study limitations

This study’s small sample size and focus on women who had participated in screening and received HPV test results, as well as inclusion only of women who spoke sufficient Spanish, limits the generalizability of its findings. So does the fact that only indigenous participants in one of the two research sites received positive HPV diagnoses. However, these qualitative findings provide important information about the perceptions of HPV and its causes that low-income Mexican women may share. Given that trends in the findings agree with results from diverse populations worldwide, these results support a call for better HPV-related education and doctor-patient communication–especially for people receiving HPV testing–worldwide. They also provide context-specific information that can be useful for improving education and screening protocols for low-income Mexican women.

## Conclusion

Education regarding the causes of HPV, and the fact that high-risk strains might but do not always inevitably cause cervical cancer in a given individual, is needed worldwide. In Mexico, EDCP protocols should include clear and comprehensive education about HPV in patients’ native languages, and clear communication and open dialogue regarding HPV testing procedures and the meaning of positive test results in relationship to cervical cancer risk and future medical appointments. Mexican sexual health care more broadly should also provide space for patients to discuss fears regarding the relationship of sexually transmitted infections to infidelity, especially in relationship to ongoing cultural debates about masculinity.

## Supporting Information

S1 Table Original Spanish Language Interview Guide. (DOCX)
