# Supplementary material for: Perceptions and Experiences of Human Papillomavirus (HPV) Infection and Testing among Low-Income Mexican Women
Source: PLoS One. 2016 May 5;11(5):e0153367. doi: 10.1371/journal.pone.0153367 (PMC4858263; doi:10.1371/journal.pone.0153367)
Supplement: S1 Table — (DOCX) [file pone.0153367.s001.docx]

**S2 Table. Original Spanish Language Interview Guide**

| *Decisión de realizar la prueba de VPH*  ¿Por qué decidió hacerse la prueba de Virus de Papiloma Humano (VPH)?  ¿Por qué pensó que era necesario hacerla?  ¿Recomendaría la prueba de VPH a una amiga, familiar o vecina? ¿Por qué?  En su opinión ¿Por qué las mujeres necesitan hacerse la prueba de VPH?  ¿Cómo convencería a alguien para que se hiciera la prueba de VPH? ¿Qué le diría para motivarla?  ¿Influye la pareja de la mujer en su decisión para realizar las pruebas de tamizaje? ¿Cómo y por qué?  ¿Por qué cree que las mujeres no desean realizar la prueba de VPH?  *La experiencia de recibir los resultados de la prueba de VPH*  ¿Cómo fue la entrega de su resultado? ¿Qué le dijeron (los profesionales de la salud) sobre su resultado?  ¿Le explicaron sus resultados de una manera fácil de entender?  ¿Qué más le hubiera gustado que le explicaran?  ¿Tuvo oportunidad de hacer preguntas? ¿Hizo alguna pregunta?  ¿Se quedó con alguna duda?  ¿Puede decirme algo sobre lo que le hubiera gustado preguntar?  ¿Cómo se sintió mientras esperaba sus resultados?  ¿Alguna cosa no le gustó cuando le entregaron sus resultados? ¿Qué sí le gustó?  ¿Qué significó para usted el resultado? ¿Qué significa un resultado positivo o negativo?  ¿Le gustaría recibir información sobre ese tema? ¿Cómo le gustaría recibir esa información?  *Comparación de la experiencia de realizar la prueba de VPH y el Papanicolaou*  ¿Vale la pena realizar las pruebas de detección del cáncer cervicouterio? ¿Por qué?  ¿Qué le hubiera gustado que fuera diferente?  ¿Le hicieron el Papanicolaou (citología cervical) y la prueba de VPH?  ¿Con qué frecuencia se realiza el Papanicolaou? ¿En dónde se lo realizan?  ¿Cuál le gusta más, la prueba de VPH o el Papanicolaou (Pap)? ¿Cree que una es mejor que la otra?  Si pudiera elegir entre la prueba de VPH y la prueba de Pap, ¿cuál elegiría? ¿Por qué?  ¿Qué no le gustó de la prueba?  ¿Piensa que hay diferencias entre las pruebas? ¿En qué son diferentes?  Cuando hicieron la prueba de Papanicolaou ¿Le preocupaba algo? ¿Cómo se sintió durante la prueba? y ¿Cuándo usted recibió los resultados?  Y ahora que se hizo la prueba de VPH, ¿le preocupa algo?  *Percepción sobre la necesidad del tamizaje*  ¿Qué mujeres cree que deben preocuparse por realizarse el Pap? ¿Cuáles mujeres cree que necesitan hacerla?  ¿Qué mujeres cree que deben preocuparse por realizarse la prueba de VPH? ¿Cuáles mujeres cree que necesitan hacerla?  Cree que la vida sexual de las mujeres, ¿tendría algo que ver con que necesiten hacerse las pruebas de detección de cáncer cervicouterino? ¿Con el Papanicolaou? ¿Con la prueba de VPH?  *Mejorar la experiencia del tamizaje*  ¿Qué debemos hacer para animar a las mujeres a hacerse la prueba de VPH?  ¿Qué podríamos hacer aquí en la unidad para que sea más fácil para las mujeres realizar la prueba de VPH?  ¿Qué podríamos hacer aquí en la unidad para que su próxima experiencia para hacer la prueba de VPH fuera mejor? ¿Qué podrían hacer para que sea más fácil para usted la próxima vez?  ¿Hay algo que no le he pedido que le gustaría compartir? |
| --- |
